# Voice outcomes following robotic transoral, robotic retroauricular, and open hemithyroidectomy

**DOI:** 10.1007/s00405-026-10566-5

**Published:** 2026-08-31

**Authors:** Chee Chean Lim, Haidatul Insyirah Sahimi, Mohd Zulkiflee bin Abu Bakar, Seo-Young Kim, Jeong-Yeon Ji, Wonjae Cha, Woo-Jin Jeong

**Affiliations:** 1 Department of Otorhinolaryngology-Head and Neck Surgery, Seoul National University Bundang Hospital, Seoul National University College of Medicine, 300 Gumi-dong, Bundang-gu, Seongnam, 13620 Korea; 2 Department of Otorhinolaryngology-Head and Neck Surgery, Faculty of Medicine, Universiti Malaya, Kuala Lumpur, Malaysia; 3 Clinical Research Department, National Heart Institute, Kuala Lumpur, Malaysia; 4 Department of Otorhinolaryngology-Head and Neck Surgery, Chung-Ang University Hospital, Chung-Ang University College of Medicine, Seoul, Republic of Korea

**Keywords:** Robotic thyroidectomy, Transoral approach, Retroauricular thyroidectomy, Voice handicap index

## Abstract

**Purpose:**

Considering the favourable prognosis of thyroid tumours, the importance of quality of life, including functional and voice outcomes, is critically important. The impact of remote-access thyroidectomy on postoperative voice function remains unclear, and direct comparative data across major approaches are limited. This study compared patient-reported and objective voice outcomes following robotic transoral (TO), robotic retroauricular (RA), and open hemithyroidectomy.

**Methods:**

This retrospective observational cohort study included patients undergoing hemithyroidectomy between January 2022 and August 2025. Voice outcomes were evaluated preoperatively and at 3 months postoperatively using the Voice Handicap Index-10 and acoustic analysis. Between-group comparisons and multivariable general linear modelling were performed to identify factors associated with postoperative voice outcomes.

**Results:**

A total of 150 patients were analysed (RA: 58; TO: 37; open: 55). Baseline VHI-10 and acoustic parameters were comparable across groups. The RA group demonstrated significantly higher postoperative VHI-10 scores and greater VHI-10 change scores compared with TO and open approaches (*p* < 0.01). No significant differences were observed in postoperative acoustic parameters, including jitter, shimmer, noise-harmonic ratio, soft phonation index, pitch range, or fundamental frequency. Female subgroup analysis demonstrated similar findings. On multivariable analysis, postoperative VHI-10 remained to be only affected by the type of surgical approach.

**Conclusion:**

All three surgical approaches showed comparable objective acoustic voice parameters. However, TO and open hemithyroidectomy were associated with better patient-perceived voice outcomes at 3 months compared with RA. When clinical conditions are comparable, TO may be considered as a viable robotic approach in patients who prioritise short-term subjective voice outcomes.

## Background

Hemithyroidectomy is increasingly being performed for benign and low-risk differentiated thyroid cancers, especially with better screening and early detection [1]. Driven by patient demand for improved cosmesis and avoidance of visible neck scars, remote access thyroidectomy (RAT) techniques have rapidly gained popularity. These techniques have demonstrated oncological safety in appropriate case selection while offering superior cosmetic results [2, 3]. It is worth noting that although many consider RAT a type of minimally invasive surgery, the actual amount of soft tissue dissection and disruption is larger to reach the thyroid gland.

Current literature largely focuses on comparing a single remote access technique (either transoral, retroauricular, or transaxillary) against open thyroidectomy, mostly confirming their non-inferiority regarding major complication rates [3–5]. However, there is a distinct lack of direct comparative data assessing the subjective and objective vocal outcomes across the major approaches within a single, standardized patient cohort. Given the distinct surgical access planes and tissue dissection associated with each technique, their impact on subtle vocal parameters is likely varied.

The study therefore aimed to compare voice outcomes following robotic transoral (TO), robotic retroauricular (RA), and open hemithyroidectomy, to support surgeons and patients in aligning operative choices with individual priorities and expectations.

## Methods

### Study population and setting

This retrospective observational cohort study included patients who underwent hemithyroidectomy via a RA, TO, or conventional open transcervical approaches, with or without central neck dissection between January 2022 to August 2025 at a single tertiary hospital. The study was approved by the Institutional Review Board of Seoul National University Bundang Hospital (B-2602-1028-101). Only patients with complete preoperative and postoperative voice evaluations were included. Surgical indications were well-differentiated thyroid cancers smaller than 2 cm and benign thyroid nodules of 5 cm or less. Patients were excluded if they demonstrated gross extrathyroidal extension, lateral compartment lymph node metastasis, distant metastasis, pre-existing laryngeal pathology, vocal cord paralysis or apparent injury to the external branch of the superior laryngeal nerve (EBSLN), age < 18 and > 70 years, history of neck irradiation, and completion thyroidectomy.

### Surgical procedure

All procedures were performed by a single surgeon (W.J.). Robotic surgeries were conducted using the da Vinci Xi surgical system (Intuitive Surgical, Inc., Sunnyvale, CA). The study design was non-randomised, and the surgical approach selection was determined according to the tumour characteristics and patient preference. The RA approach was preferentially selected for tumours located near the posterior thyroid capsule. Written informed consent was obtained from all patients, understanding the risks, benefits and possibility for conversion to open surgery.

Intraoperatively, functional integrity of the recurrent laryngeal nerve (RLN) and EBSLN was monitored using the NIM 3.0 nerve monitoring system (Medtronic, Jacksonville, FL). The RLN was visually identified and confirmed using nerve stimulation. EBSLN preservation was confirmed by stimulation along its course with the presence of cricothyroid muscle contraction before and after securing the superior thyroidal vessels.

Conventional open transcervical hemithyroidectomy was performed with a standard transverse incision. Strap muscles were preserved without transection in all cases.

For the RA approach, an incision was made along the occipital hairline, and the skin flap was elevated over the sternocleidomastoid (SCM) until its anterior border. The greater auricular nerve was traced and preserved during flap elevation. The omohyoid was identified, and the strap muscles were retracted using an external retractor. A 30-degree down-viewing endoscope was positioned in the central robotic arm 3. Two Maryland bipolar fenestrated forceps were placed in each of the non-dominant arms 1 and 2, and a monopolar spatula was positioned in the dominant arm. Further details regarding robotic docking and placement of the four robotic arms have been described previously [6]. The thyroid isthmus was divided, and the superior pole was dissected from the inferior constrictor muscle. The superior thyroid vessels were secured, and the superior parathyroid gland was carefully preserved. The thyroid gland was then retracted medially to allow identification and preservation of the RLN and the inferior parathyroid gland.

For the TO approach, a 1.5–2 cm central mucosal incision was created at the lower lip frenulum, and two lateral incisions were made close to the oral commissures to avoid mental nerve injury. Subplatysmal working space was created using blunt dissection extending from the mandible to the central neck. Three trocars were inserted into the working space, to facilitate placement of a central 30-degree endoscope and lateral robotic instruments (Maryland bipolar fenestrated forceps and monopolar spatula). An additional robotic arm with a Maryland bipolar fenestrated forceps was introduced via a left axillary port. Following docking, a midline strap muscle incision was created, and dissection of the thyroid was performed. Similarly, identification and dissection of the RLN was assisted by intraoperative nerve monitoring. The specimen was retrieved using an endo-bag to avoid tumour spillage. Strap muscles were reapproximated with V-Loc sutures, and the vestibular incisions were approximated with resorbable sutures.

### Voice assessment

Rigid laryngoscopy was performed preoperatively, on postoperative day 1, and at 3 months to evaluate RLN and EBSLN integrity. Patient-reported voice evaluation using the Voice Handicap Index-10 (VHI-10) questionnaire was performed preoperatively and at 3 months after surgery.

Objective voice tests using the Computerized Speech Lab (Model 4500b; KayPENTAX, Lincoln Park, NJ) included pre- versus 3 months post-treatment voice range profile (VRP), multidimensional voice program (MDVP), and the maximum phonation time (MPT). VRP provided measurements of the frequency range (Hz and semitones), highest frequency (F-high, Hz), lowest frequency (F-low, Hz), and intensity range (dB). MDVP parameters included fundamental frequency (F0, Hz), jitter (%), shimmer (%), and noise to harmonic ratio (dB), and soft phonatory index (SPI).

### Statistical analyses

Baseline clinicopathologic characteristics were compared across the three surgical approaches. Continuous variables were compared using one-way analysis of variance (ANOVA) or Kruskal-Wallis test, as appropriate. Categorical variables were analysed using chi-square test or Fisher’s exact test.

Within-group comparisons were performed using a paired t-test or Wilcoxon signed-rank test, while between-group comparisons were analysed using ANOVA or Kruskal-Wallis test, as appropriate. Lastly, multivariable general linear modelling was performed to evaluate factors associated with postoperative VHI-10 scores. Thyroidectomy approach, alongside clinicopathologic and operative variables, were included as covariates in the model. Statistical significance was defined as a p value of less than 0.05.

## Results

### Clinicopathological characteristics

A total of 284 patients who had undergone hemithyroidectomy during the study period were screened for inclusion. Thirty-eight patients were excluded according to the pre-defined exclusion criteria, including two cases of postoperative vocal cord paralysis (one each from the TO group and the RA group). An additional 96 patients were excluded owing to missing VHI-10 questionnaires, incomplete acoustic data, or loss to follow-up. Ultimately, 150 patients successfully completed all scheduled follow-up assessments, including both the VHI-10 questionnaire and acoustic analysis.

Baseline characteristics differed between groups, exhibiting baseline group variability in which the robotic approaches, particularly the RA group, were more frequently performed in younger females with lower body mass index (BMI), while oncologic variables were largely comparable (Table 1).

**Table 1:** Clinicopathologic characteristics of the robotic retroauricular, robotic transoral and open hemithyroidectomy groups

Characteristics	Robotic retroauricular (*N* = 58)	Robotic transoral (*N* = 37)	Open transcervical (*N* = 55)	*p* value
Gender				
Male	8 (13.8)	11 (29.7)	21 (38.2)	0.012*
Female	50 (86.2)	26 (70.3)	34 (61.8)	
Age				
Mean ± SD	41.5 ± 10.8	41.1 ± 9.8	48.4 ± 14.6	0.003*
BMI (kg/m^2^)				
Median (Q1, Q3)	22.8 (20.8, 25.0)	23.7 (21.6, 27.1)	26.3 (22.7, 28.8)	0.009*
Smoking status				
Non-smoker	56 (96.6)	35 (94.6)	47 (85.5)	0.075
Smoker	2 (3.4)	2 (5.4)	8 (14.5)	
Alcohol				
Non-user	40 (69.0)	31 (83.8)	38 (69.1)	0.217
User	18 (31.0)	6 (16.2)	17 (30.9)	
T- staging				
pT1	41 (70.7)	34 (91.9)	44 (80.0)	0.304
pT2	7 (12.1)	2 (5.4)	5 (9.1)	
pT3	4 (6.9)	1 (2.7)	3 (5.5)	
N staging				
N0	51 (87.9)	35 (94.6)	32 (58.2)	< 0.001*
N1a	1 (1.7)	2 (5.4)	20 (36.4)	
Tumor				
Benign	6 (10.3)		3 (5.5)	0.115
Malignant	52 (89.7)	37 (100.0)	52 (94.5)	
Nodule size				
Median (Q1, Q3)	0.7 (0.6, 1.9)	0.8 (0.5, 1.0)	1.0 (0.6, 1.9)	0.060
Multiplicity				
No	44 (78.6)	30 (83.3)	38 (71.7)	0.418
Yes	12 (21.4)	6 (16.7)	15 (28.3)	
Operative time (min)				
Mean ± SD	179.5 ± 41.1	172.0 (143.0, 193.0)	109.0 (88.0, 132.0)	< 0.001*
Hematoma				
No	58 (100.0)	37 (100.0)	55 (100.0)	NA
Vocal cord palsy (excluded)	1 (1.7)	1 (2.6)	0	0.521

Data are presented as n (%), mean ± SD, or median (Q1, Q3), as appropriate

Vocal cord palsy was excluded from the analysis

Significant values indicated with *

### Overall VHI-10 and acoustic outcomes

**Fig. 1:**
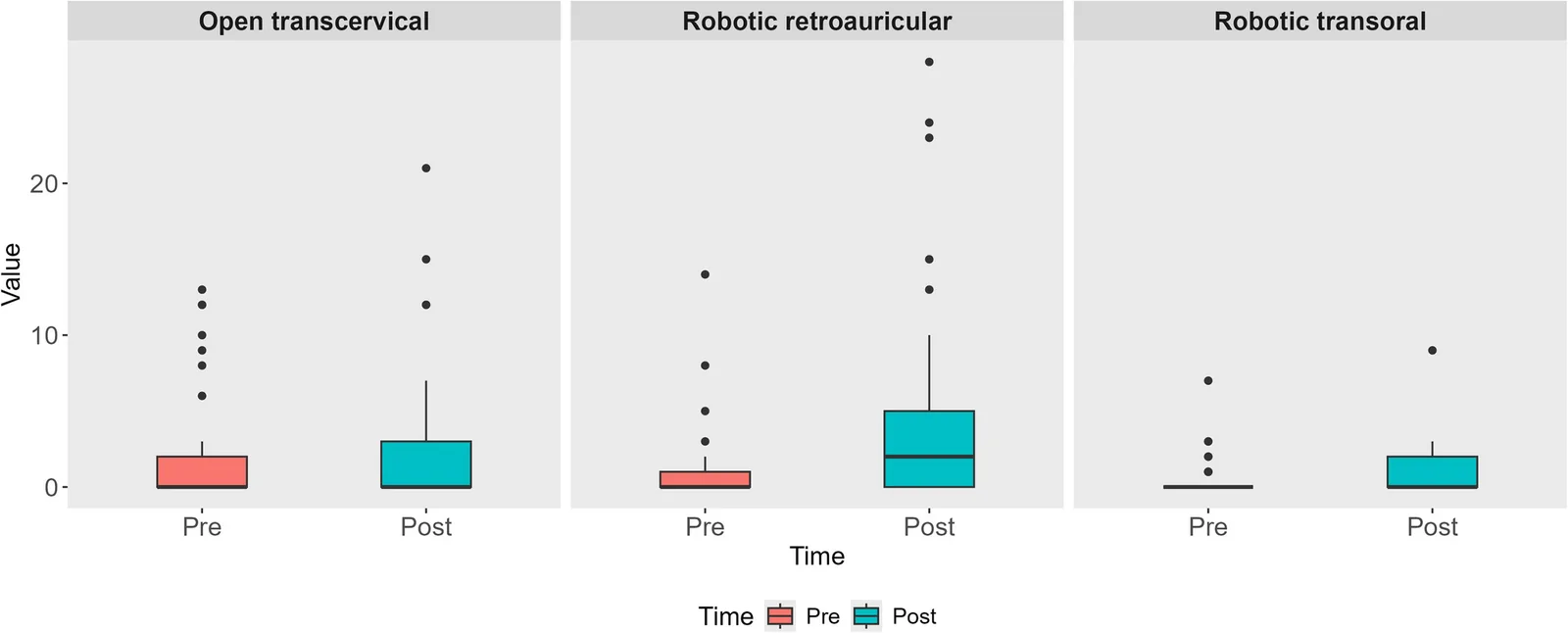
Line plot of preoperative and postoperative VHI-10 scores for open transcervical, robotic retroauricular and robotic transoral

On 3-month laryngoscopy, all patients showed unremarkable vocal fold mobility and no mucosal lesions related to phonation, except for one patient in the RA group who showed equivocal vocal fold mobility, likely related to tongue traction during rigid laryngoscopy. Preoperative VHI-10 scores were low and comparable across groups. Postoperatively, the RA approach demonstrated significantly higher VHI-10 scores (Fig. 1) and greater VHI-10 change scores compared with the TO and open approaches (Table 2). VHI-10 scores were low at presentation across all patients, and most remained well within normal limits throughout the study period. Although the RA group showed a postoperative elevation in VHI-10, the magnitude of change was small and the scores remained within normal limits.

**Table 2:** Comparison of preoperative and postoperative VHI-10 and acoustic parameters in the robotic retroauricular, robotic transoral and open approaches

Characteristics	Robotic retroauricular (*N* = 58) Median (Q1, Q3)	*P* value (pre–post)	Robotic transoral (*N* = 37), Median (Q1, Q3)	*P* value (pre–post)	Open transcervical (*N* = 55) Median (Q1, Q3)	*P* value (pre–post)	*P* value (between groups)
VHI*							
Pre	0.00 (0.00, 1.00)	< 0.001*	0.00 (0.00, 0.50)	0.212	0.00 (0.00, 2.00)	0.169	0.209
Post	2.00 (0.00, 5.50)		0.00 (0.00 (2.00)		0.00 (0.00, 3.00)		0.003*
Δ VHI	0.50 (0.00, 4.25)		0.00 (0.00, 2.00)		0.00 (0.00, 1.00)		0.009*
F0							
Pre	198.05 (167.75, 223.15)	0.076	207.30 (161.45, 234.70)	0.255	186.1 (121.20, 215.60)	0.624	0.046*
Post	200.80 (179.00, 229.10)		204.80 (135.85, 235.75)		183.80 (118.5, 220.20)		0.049*
Δ F0	9.45 (-10.85, 22.98)		-5.30 (-18.4, 8.25)		-2.30 (-12.80, 15.10)		0.113
Jitter							
Pre	0.64 (0.35, 1.45)	0.185	0.48 (0.33, 0.86)	0.525	0.62 (0.37, 1.09)	0.269	0.322
Post	0.49 (0.34, 0.93)		0.47 (0.38, 0.89)		0.62 (0.40, 0.89)		0.811
Δ Jitter	-0.06 (-0.60, 0.21)		0.03 (-0.14, 0.24)		-0.02 (-0.47, 0.25)		0.472
Shimmer							
Pre	2.96 (2.30, 4.24)	0.021*	2.84 (2.23, 3.62)	0.474	3.17 (2.19, 4.18)	0.384	0.581
Post	2.56 (1.86, 3.35)		3.02 (2.26, 3.95)		3.21 (1.97, 3.94)		0.108
Δ Shimmer	-0.20 (-1.38, 0.43)		0.04 (-0.62, 0.81)		-0.30 (-1.32, 1.24)		0.141
NHR							
Pre	0.13 (0.11, 0.14)	0.788	0.12 (0.11, 0.13)	0.111	0.13 (0.11, 0.14)	0.408	0.109
Post	0.12 (0.11, 0.14)		0.12 (0.12, 0.14)		0.13 (0.11, 0.14)		0.420
Δ NHR	0.005 (-0.019, 0.013)		0.008 (-0.011, 0.026)		-0.003 (-0.022, 0.019)		0.147
SPI							
Pre	11.36 (7.76, 16.04)	0.399	9.25 (5.10, 17.01)	0.613	11.90 (7.70, 16.60)	0.339	0.270
Post	11.30 (7.52, 17.49)		9.39 (6.51, 15.83)		11.05 (7.08, 15.81)		0.383
Δ SPI	0.41 (-4.14, 6.01)		0.85 (-2.91, 3.67)		-0.78 (-5.76, 3.93)		0.447
F high							
Pre	415.30 (311.10, 523.20)	0.972	392.00 (311.10, 466.10)	0.480	369.90 (293.60, 493.80)	0.085	0.684
Post	392.00 (329.60, 493.80)		369.90 (277.10, 427.65)		329.60 (261.60, 466.10)		0.134
Δ F High	0 (-67.58, 81.20)		-18.50 (-90.95, 55.50)		-40.30 (-93.50, 45.40)		0.439
F low							
Pre	164.80 (138.58, 185.00)	0.111	155.50 (110.00, 196.00)	0.088	155.50 (103.80, 174.60)	0.225	0.091
Post	174.60 (144.75, 187.75)		164.80 (131.65, 196.00)		138.50 (103.80, 185.00)		0.034*
Δ F low	0.05 (-9.95, 17.63)		9.30 (-10.70, 22.10)		4.30 (-8.30, 12.70)		0.649
F range (Hz)							
Pre	248.25 (152.38, 340.40)	0.790	230.30 (143.90, 287.70)	0.281	223.10 (146.30, 356.10)	0.079	0.708
Post	227.10 (164.05, 316.50)		184.90 (117.65, 268.65)		195.30 (157.80, 270.10)		0.227
Δ F range (HZ)	-0.95 (-80.30, 79.15)		-27.90 (-100.45, 46.60)		-42.20 (-105.80, 36.60)		0.576
F range (ST)							
Pre	16.00 (12.75, 20.00)	0.575	15.00 (12.00, 20.50)	0.158	16.00 (13.00, 23.00)	0.192	0.571
Post	15.00 (12.00, 18.25)		13.00 (11.50, 17.00)		16.00 (13.00, 19.00)		0.071
Δ F range (ST)	0.00 (-4.25, 3.25)		0.00 (-5.00, 1.50)		-2.00 (-5.00, 3.00)		0.676
Intensity range							
Pre	12.50 (7.75, 19.00)	0.556	13.00 (8.00, 16.00)	0.431	14.00 (10.00, 18.00)	0.693	0.384
Post	12.00 (9.00, 17.00)		14.00 (8.00, 19.00)		14.00 (10.00, 17.00)		0.747
Δ Inten range	0.00 (-5.25, 3.25)		1.00 (-4.00, 5.00)		0.00 (-6.00, 4.00)		0.550
MPT							
Pre	14.00 (11.27, 18.49)	0.004*	14.45 (10.95, 17.04)	0.988	14.44 (11.09, 17.57)	0.311	0.830
Post	16.79 (12.99, 19.35)		13.22 (11.06, 17.52)		14.77 (12.12, 17.59)		0.033*
Δ MPT	1.73 (-0.84, 4.05)		-0.50 (-2.76, 3.33)		0.73 (-2.42, 4.28)		0.209

Values are expressed as median (Q1, Q3). Δ = Post – Pre

VHI voice handicap index, F0 fundamental frequency, NHR noise harmonic ratio, SPI soft phonatory index, F frequency, MPT maximum phonation time

Significant values indicated with *

Although intergroup differences in fundamental frequency were observed at baseline and postoperative assessment, change scores did not differ significantly across groups. Acoustic perturbation measures including jitter, shimmer, NHR, and SPI, showed no significant postoperative or change-score differences between the thyroidectomy approaches.

Pitch-related parameters showed minor intergroup variation in postoperative F-low values, but no significant differences in pitch range or intensity range were observed. MPT increased significantly after surgery in the RA group, but the degree of change was not statistically significant. Overall, no significant postoperative changes were observed in the acoustic measures, with the exception of the VHI-10 changes described above.

### Female subgroup analysis

Within female patients, baseline voice measures were comparable except for higher preoperative NHR in the open approach. Postoperatively, VHI-10 scores remained significantly higher in the RA group. Similarly, the change in VHI-10 scores differed significantly. Although NHR change differed significantly between groups, absolute postoperative NHR values were comparable. Overall, objective acoustic outcomes remained similar across approaches in female patients (Table 3).

**Table 3:** Female Subgroup comparison of VHI-10 and acoustic parameters

Characteristics	Robotic retro auricular (*N* = 50) Median (Q1, Q3)	*P* value (pre–post)	Robotic transoral (*N* = 26), Median (Q1, Q3)	*P* value (pre–post)	Open transcervical (*N* = 34) Median (Q1, Q3)	*P* value (pre–post)	*P* value (between groups)
VHI*							
Pre	0.00 (0.00, 1.00)	< 0.001*	0.00(0.00, 1.00)	0.295	0.00 (0.00, 2.00)	0.549	0.438
Post	2.50 (0.00, 7.25)		0.00 (0.00, 2.00)		0.50 (0.00, 3.00)		0.004*
Δ VHI	2.00 (0.00, 5.25)		0.00 (-1.00, 2.00)		0.00 (0.00, 0.25)		0.005*
F0							
Pre	202.70 (182.60, 230.75)	0.118	220.05 (191.80, 258.05)	0.431	204.25 (187.40, 237.68)	0.771	0.100
Post	207.80 (198.63, 20.63)		225.30 (200.15, 242.38)		204.35 (189.05, 240.55)		0.379
Δ F0	7.75 (-10.85, 26.83)		-11.10 (-19.75, 17.90)		-2.20 (-19.08, 17.48)		0.260
Jitter							
Pre	0.71 (0.35, 1.57)	0.081	0.51 (0.33, 0.95)	0.094	0.64 (0.36, 1.14)	0.925	0.372
Post	0.49 (0.36, 0.89)		0.53 (0.42, 0.99)		0.71 (0.45, 1.00)		0.388
Δ Jitter	-0.09 (-0.72, 0.20)		0.06 (-0.04, 0.39)		0.08 (-0.44, 0.42)		0.130
Shimmer							
Pre	3.02 (2.31, 4.66)	0.018*	2.89 (2.12, 3.59)	0.381	3.05 (2.16, 4.20)	0.510	0.623
Post	2.58 (1.92, 3.35)		3.03 (2.12, 3.88)		3.43 (1.97, 4.03)		0.174
Δ Shimmer	-0.32 (-1.54, 0.43)		0.07 (-0.42, 0.70)		-0.38 (-1.32, 1.33)		0.103
NHR							
Pre	0.12 (0.11, 0.14)	0.676	0.11 (0.10, 0.13)	0.029*	0.13 (0.11, 0.15)	0.046*	0.031*
Post	0.12 (0.11, 0.14)		0.12 (0.12, 0.13)		0.12 (0.11, 0.14)		0.839
Δ NHR	0.005 (-0.020, 0.013)		0.010 (-0.001, 0.028)		-0.011 (-0.035, 0.013)		0.014*
SPI							
Pre	10.96 (7.07, 15.21)	0.188	8.60 (5.05, 16.93)	0.469	11.99 (8.27, 15.10)	0.180	0.301
Post	11.00 (7.26, 17.21)		9.70 (6.41, 15.72)		10.61 (6.23, 14.82)		0.621
Δ SPI	1.34 (-3.26, 6.01)		1.05 (-2.14, 3.61)		-2.43 (-6.43, 3.30)		0.172
F high							
Pre	427.65 (349.20, 530.98)	0.773	415.30 (344.30, 466.13)	0.294	415.30 (344.30, 539.23)	0.200	0.821
Post	415.30 (349.20, 523.20)		369.90 (329.60, 453.45)		415.30 (329.60, 437.03)		0.463
Δ F High	0.00 (-75.50, 79.10)		-28.70 (-99.12, 54.65)		-42.85 (-82.18, 45.40)		0.629
F low							
Pre	155.50 (174.30, 185.00)	0.102	185.00 (155.50, 196.00)	0.092	174.60 (155.50, 196.00)	0.330	0.344
Post	179.80 (164.80, 196.00)		185.00 (164.80, 196.00)		174.60 (155.50, 96.00)		0.335
Δ F low	4.40 (-9.95, 18.55)		9.30 (-10.55. 34.00)		9.55 (-13.55, 19.10)		0.713
F range (Hz)							
Pre	259.70 (191.30, 351.03)	0.581	238.20 (152.95, 284.45)	0.137	234.60 (173.98, 379.60)	0.188	0.543
Post	235.25 (186.75, 327.65)		205.10 (149.65, 265.00)		246.10 (162.35, 303.18)		0.233
Δ F range (HZ)	-6.15 (-103.45, 79.15)		-47.75 (-105.75, 46.10)		-49.90 (-97.18, 36.60)		0.710
F range (ST)							
Pre	16.00 (12.75, 20.25)	0.360	13.50 (11.00, 17.25)	0.068	15.50 (11.00, 18.25)	0.245	0.353
Post	15.00 (12.00, 18.25)		12.50 (10.50, 16.00)		14.50 (10.75, 18.00)		0.078
Δ F range (ST)	-0.50 (-5.00, 3.00)		-2.00 (-6.25, 1.25)		-2.50 (-5.00, 1.50)		0.688
Inten range							
Pre	13.00 (8.00, 19.25)	0.268	12.00 (7.75, 16.00)	0.367	12.0 (9.75, 16.25)	0.318	0.826
Post	12.00 (9.00, 17.00)		13.00 (8.00, 18.00)		12.00 (10.00, 15.25)		0.861
Δ Inten range	-0.50 (-5.25, 3.00)		1.00 (-3.00, 4.25)		0.00 (-6.25, 2.00)		0.319
MPT							
Pre	13.79 (11.27, 16.90)	0.004*	14.21 (10.99, 16.10)	0.611	12.72 (10.60, 16.51)	0.555	0.657
Post	16.29 (12.57, 18.48)		12.19 (10.73, 15.06)		14.04 (10.18, 17.00)		0.015*
Δ MPT	1.73 (-0.74, 3.60)		-0.15 (-3.19, 2.21)		1.06 (-2.38, 3.78)		0.132

Values are expressed as median (Q1, Q3). Δ = Post – Pre

VHI voice handicap index, F0 fundamental frequency, NHR noise harmonic ratio, SPI soft phonatory index, F frequency, MPT maximum phonation time

Significant values indicated with *

### Factors associated with postoperative VHI-10

Multivariable general linear modelling demonstrated that the thyroidectomy approach was the only independent predictor of postoperative VHI-10 scores. Patients undergoing RA hemithyroidectomy showed significantly higher VHI-10 scores compared to the TO approach. No significant differences were observed between RA and open or between TO and open approaches.

Demographic, tumour-related, and operative variables were not significantly associated with postoperative VHI-10 scores (Table 4).

**Table 4:** General Linear Model for Postoperative VHI-10 scores. Tests of Between-Subjects Effects

Source	Type III Sum of Squares		df	Mean Square	F	Sig.
Corrected Model	385.647^a^		15	25.710	1.294	0.215
Intercept	26.326		1	26.326	1.325	0.252
Thyroidectomy groups	137.466		2	68.733	3.461	0.034
Benign2	20.214		2	10.107	0.509	0.602
T- staging	52.258		2	26.129	1.316	0.272
N- staging	8.507		1	8.507	0.428	0.514
Smoking	47.535		1	47.535	2.393	0.124
Alcohol	3.381		1	3.381	0.170	0.681
Gender	27.895		1	27.895	1.404	0.238
Multiplicity	6.103		1	6.103	0.307	0.580
Age	16.548		1	16.548	0.833	0.363
BMI (kg/m2)	2.833		1	2.833	0.143	0.706
Tumor nodule size	0.401		1	0.401	0.020	0.887
Operative time (min)	29.964		1	29.964	1.509	0.222
(I) Thyroidectomy groups	(J) Thyroidectomy groups	Mean Difference (I-J)	Std. Error	Sig.	95% CI Lower	95% CI Upper
Robotic retro auricular	Robotic transoral	2.536*	1.009	0.013*	0.539	4.534
Robotic retro auricular	Open transcervical	2.207	1.317	0.096	-0.400	4.814
Robotic transoral	Robotic retro auricular	-2.536*	1.009	0.013*	-4.534	-0.539
Robotic transoral	Open transcervical	-0.329	1.320	0.803	-2.942	2.283
Open transcervical	Robotic retro auricular	-2.207	1.317	0.096	-4.814	0.400
Open transcervical	Robotic transoral	0.329	1.320	0.803	-2.283	2.942

^a^R² = 0.135, Adjusted R² = 0.031

The mean difference is significant at *p* < 0.05 (Least Significant Difference method)

## Discussion

This study utilizes a single institutional, single-surgeon cohort to provide a controlled comparison of voice outcomes across RA, TO and open hemithyroidectomy. The study benefits from a standardized longitudinal protocol, with all patients undergoing preoperative and 3-month postoperative voice evaluations. While objective acoustic parameters remained preserved across surgical approaches, the RA approach was associated with significantly higher patient-perceived voice handicap.

It is well recognised that surgeon familiarity, training background and institutional expertise strongly influence the choice of surgical approach, particularly for robotic techniques. Such preferences are unlikely to change especially when oncologic safety and operative efficiency are preserved. The primary intent of the study is therefore not to advocate for or against a specific approach, but rather to provide clinical outcomes that may facilitate more informed perioperative counselling and shared decision-making.

The significant differences observed between the groups, specifically the younger female with lower body mass index (BMI) in the robotic approaches, reflect well-known selection biases inherent in non-randomized surgical studies, where these patients frequently opt for or are selected for RAT approaches [3]. Despite longer operative times, our robotic approaches demonstrated comparable safety profiles, consistent with existing literature [2–5, 7]. It should be noted that while these robotic procedures can be safely performed with minimal morbidity by high-volume, experienced surgeons, they could yield a potentially inferior outcome if performed by an inexperienced surgeon.

Postoperative VHI-10 ratings generally did not differ significantly across studies comparing between individual robotic approaches against open transcervical [8–10]. Nonetheless, a few studies have shown that, especially in female patients, TO and RA approaches might produce broader pitch ranges and higher maximum frequencies in the postoperative period [7, 9–11]. This may reflect subtle improvements in the objective voice acoustics despite similar overall patient-perceived impairment. However, current evidence on voice outcomes between the types of robotic approaches is limited and characterized by small sample size, variable follow-up, and inconsistent outcome measures. Therefore, conclusions about the superiority of remote-access approaches in voice outcomes must be drawn with caution.

The RA technique requires extensive subplatysmal flap elevation and prolonged strap muscle retraction. This can lead to subsequent edema, fibrosis and fatigue of the overlying soft tissues and strap muscles causing postoperative cervical stiffness and altered extralaryngeal tension [12, 13]. This increases the risk of post-thyroidectomy dysphonia even in the absence of obvious nerve injury as patients perceive this as an increased strain in their vocal efforts [14]. Furthermore, the full course of the EBSLN, including its origin, lies within the working space and may be fully exposed and subjected to traction during dissection. In addition, the retractor used for RA approach may exert considerable mechanical force on the surrounding tissues [15]. While these factors may contribute to subjective voice changes, this remains speculative as they were not directly evaluated in the present study.

Finally, the manipulation or transection of the greater auricular nerve and other superficial cervical sensory nerves often results in anesthesia or paresthesia over the earlobe and neck area. This discomfort and self-awareness may lead to a perceived deficiency in the Functional and Emotional subscales, especially among female patients who could be more sensitive to changes in neck sensation and cosmesis.

The discordance between patient-reported voice impairment and objective acoustic preservation is clinically important. Acoustic analyses primarily assess vocal fold vibration in controlled settings, while the VHI-10 measures perceived vocal effort and functional communication. The surgical method was independently correlated with postoperative VHI-10 ratings, affirming its role as a determinant of patient-perceived voice outcomes. The observed increase in MPT within the RA group may reflect compensatory hyperfunctional phonation instead of improved voice quality [16]. Collectively, these findings suggest that RA approach may primarily influence extralaryngeal biomechanics rather than intrinsic vocal fold function.

This study has several limitations. First, the retrospective and non-randomised design introduces selection bias. Baseline demographic differences between groups may also confound functional outcomes despite multivariable adjustment. Second, the modest sample size, especially in the TO group, may limit the statistical power for subgroup analysis. Thirdly, the follow-up voice assessments were limited to 3 months, which may not capture the long-term voice outcomes. Fourthly, no perceptual voice assessment (e.g., GRBAS or CAPE-V) is included. Lastly, because only small tumours (< 2 cm) or benign nodules were included, this technique comparison is not applicable to larger and more extensive tumour presentations.

## Conclusion

Objective voice parameters were comparable across all three surgical approaches. However, robotic retroauricular (RA) hemithyroidectomy was associated with increased patient-reported voice handicap at 3 months postoperatively, despite the preservation of acoustic parameters. These findings should be interpreted strictly as short-term outcomes. It should also be emphasised that, although the subjective scores increased in the RA group, the VHI-10 scores remained well within normal limits across all groups. For patients with specific short-term voice-related needs, this finding may be considered during counselling; however, it should be interpreted cautiously and not regarded as evidence of superiority of one surgical approach over another.
